# Synthesis, Molecular Docking and β-Glucuronidase Inhibitory Potential of Indole Base Oxadiazole Derivatives

**DOI:** 10.3390/molecules24050963

**Published:** 2019-03-08

**Authors:** El Hassane Anouar, Moustapha Eid Moustapha, Muhammad Taha, Mohammed H. Geesi, Zeinab R. Farag, Fazal Rahim, Noor Barak Almandil, Rai Khalid Farooq, Muhammad Nawaz, Ashik Mosaddik

**Affiliations:** 1 Department of Chemistry, College of Science and Humanities, Prince Sattam bin Abdulaziz University, P.O. Box 83, Al Kharj 11942, Saudi Arabia; m.moustapha@psau.edu.sa (M.E.M.); m.geesi@psau.edu.sa (M.H.G.); 2 University Central Laboratory, College of Science and Humanities, Prince Sattam bin Abdulaziz University, P.O. Box 83, Al Kharj 11942, Saudi Arabia; 3 Department of Clinical Pharmacy, Institute for Research and Medical Consultations (IRMC), Imam Abdulrahman Bin Faisal University, P.O. Box 1982, Dammam 31441, Saudi Arabia; nbalmandil@iau.edu.sa (N.B.A.); amosaddik@iau.edu.sa (A.M.); 4 Chemistry Department, Faculty of Science, Fayoum University, Fayoum 63514, Egypt; zrf00@fayoum.edu.eg; 5 Department of Chemistry, Hazara University, Mansehra, Khyber Pakhtunkhwa 21300, Pakistan; fazalstar@gmail.com; 6 Department of Neuroscience Research, Institute for Research and Medical Consultations (IRMC), Imam Abdulrahman Bin Faisal University, P.O. Box 1982, Dammam 31441, Saudi Arabia; rkfarooq@iau.edu.sa; 7 Department of Nano-Medicine Research, Institute for Research and Medical Consultations (IRMC), Imam Abdulrahman Bin Faisal University, P.O. Box 1982, Dammam 31441, Saudi Arabia; mnnmuhammad@iau.edu.sa

**Keywords:** synthesis, indole, oxadiazole, β-glucuronidase, molecular docking, SAR

## Abstract

β-glucuronidase is a lysosomal glycosidase enzyme which catalyzes the extracellular matrix of cancer and normal cells and the glycosaminoglycans of the cell membrane, which is important for cancer cell proliferation, invasion, and metastasis. Liver cancer, colon carcinoma, and neoplasm bladder are triggered by the increase of the level of β-glucuronidase activity. The most valuable structures are indole and oxadiazole which has gain immense attention because of its pharmacological behavior and display many biological properties. Twenty-two (**1**–**22**) analogs of indole based oxadiazole were synthesized and screened for their inhibitory potential against β-glucuronidase. Majority of the compounds showed potent inhibitory potential with IC_50_ values ranging between 0.9 ± 0.01 to 46.4 ± 0.9 µM, under positive control of standard drug d-saccharic acid 1,4 lactone (IC_50_ = 48.1 ± 1.2 µM). Structural activity relationship (SAR) has been established for all synthesized compounds. To shed light on molecular interactions between the synthesized compounds and β-glucuronidase, **1**, **4**, and **6** compounds were docked into the active binding site of β-glucuronidase. The obtained results showed that this binding is thermodynamically favorable and β-glucuronidase inhibition of the selected compounds increases with the number of hydrogen bonding established in selected compound-β-glucuronidase complexes.

## 1. Introduction

β-glucuronidase is an enzyme that catalyzes the extracellular matrix of cancer and normal cells and also catalyzes glycosaminoglycans of the cell membrane. This catalysis is important for cancer cell proliferation, invasion, and metastasis [1,2]. Liver cancer, colon carcinoma and neoplasm bladder are caused by the increase level of β-glucuronidase activity [3,4,5,6]. β-glucuronidase has been used as an important tool in the detection of real time monitoring, clinical therapies, diagnosis in early stage, and used in tumor marker [7,8]. High level of β-glucuronidase is associated with various diseases including epilepsy [9] and other physiological disorder. β-glucuronidase increase level cause liver damage which is a major issue worldwide [10]. The deficiency of GUS in the human body causes mucopolysaccharidosis type VII (MPSVII, known as Sly syndrome) due to the accumulation of glycosoaminoglycans containing glucuronic acid residues, leading to lysosomal storage in the brain [11,12]. It is very important to inhibit this enzyme to prevent such physiological disorder. The heterocyclic compounds cover one of the important fields in medicinal chemistry. They occupy an important position in the structures of a variety of natural products. One of the most valuable structures is indole which has gain immense attention because of its pharmacological behavior and has been considered a vital scaffold [13,14]. A number of indole-based compounds have been reported for their potential anticancer activities [15]. Hydrazono-indole analogs have been reported to induce apoptosis [16], and to interact with tubulin [17,18,19].

On the other hand, compounds that contained oxadiazole ring in their structures also display many biological properties including anticancer [20], cytotoxic and antimicrobial [21], anticonvulsant [22], antiepileptic [23], anti-TB [24], and anti-allergic activities [25]. Both structural moieties are potent and their combination has been reported to have enhanced biological activity [26,27].

Our research group has reported various heterocyclic compounds classes as potent inhibitors of β-glucuronidase [28,29,30,31,32,33,34,35]. Here, we report the synthesis and the β-glucuronidase inhibition of a series of 22 indole-based oxadiazole derivatives.

## 2. Results and Discussion

### 2.1. Chemistry

**Scheme 1:**
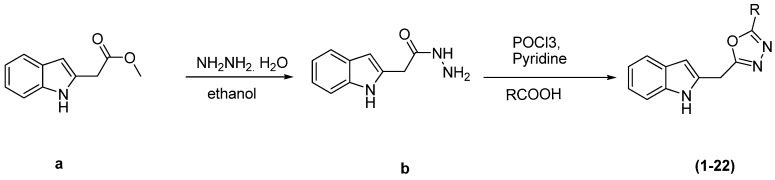
Synthesis of (**1**–**22**) indole analogs.

A series of indole based 2,5-disubstituted-1,3,4-oxadiazoles (**1**–**22**, Scheme 1) was synthesized affording to literature protocol [36]. The methyl 2-(1*H*-indol-2-yl) acetate (**a**) was refluxed with ethanolic hydrazine solution for six hours to provide 2-(1*H*-indol-2-yl) acetohydrazide (**b**) followed by reflux 2-(1*H*-indol-2-yl) acetohydrazide (**b**) was formerly treated with diverse aromatic carboxylic acids in POCl_3_ to achieve indole based oxadiazoles (**1**–**22**). Characterization of all synthesized compounds was performed using ^1^H NMR, ^13^C NMR, and mass spectra which originated to be in complete arrangement with the projected structures.

### 2.2. Biological Activity

In this study, we have carried out the synthesis of indole based oxadiazole analogs (**1**–**22**) and their evaluation against β-glucuronidase. Majority of the compounds showed potent inhibitory potential with IC_50_ values ranging from 0.9 ± 0.01 to 46.4 ± 0.9 µM when compared with the positive control d-saccharic acid 1,4 lactonec (IC_50_ = 48.1 ± 1.2 µM).

Structural activity relationship has been established for all compounds. Compound **6** having at the *ortho*-*meta* position the dihydroxyl groups on the phenyl ring was found the most active analog among the series. The greater inhibitory potential of this compound may be due to the position as well as the vicinity of the dihydroxy groups.

If we compare analog **6** with other dihydroxy analogs i.e., analog **5** (IC_50_ = 11.4 ± 0.30 µM), analog **7** (IC_50_ = 1.2 ± 0.01 µM) and analog **8** (IC_50_ = 7.2 ± 0.10 µM), it was found that the Compound **6** is much more potent. This higher activity of analog **6** is seems due to the hydroxyl groups position on the phenyl ring which is *ortho*-*meta*. Additionally, the activity may also be enhanced when both hydroxyl groups are at the vicinal position. If we compare the dihydroxy analogs with mono-hydroxy analogs i.e., analog **3** (IC_50_ = 6.2 ± 0.2 µM), analog **4** (IC_50_ = 17.90 ± 0.4 µM), and analog **9** (IC_50_ = 11.0 ± 0.4 µM), the dihydroxy analogs were found to be most superior. The greater potential indicates that position and number of the hydroxyl groups on phenyl ring play a vital role in the inhibitory potential.

Comparing the nitro substituted analogs **10** (IC_50_ = 40.0 ± 0.7 µM), **11** (NAb) and **13** (IC_50_ = 37.3 ± 0.7 µM), the *ortho* and *para* substituted analogs **10** and **12** were found active while analog **11** was found inactive. This indicates that the position of the nitro group at *ortho* and *para* positions enhances the activity rather than *meta* position.

If we compared the *o*-fluoro substituted analog **18** (IC_50_ = 5.0 ± 0.1 µM) with *m*-fluoro analog **19** (IC_50_ = 10.5 ± 0.2 µM) and *p*-fluoro substituted analog **20** (IC_50_ = 17.0 ± 0.4 µM), analog **18** was found more superior and the reason for this greater potential is that *ortho* substituted analog is more favorable for inhibition of the enzyme. Similarly, the *ortho* substituted chloro analog **21** with IC_50_ = 6.0 ± 0.2 µM showed more potency when compared with *meta* and *para* substituted analogs **15** (IC_50_ = 34.6 ± 0.7 µM) and **22** (IC_50_ = 22.2 ± 0.5 µM), respectively. This greater inhibition of Compound **21** is attributed to the position of chloro group at *ortho* position. Compounds containing pyridine in their structures—i.e., Compounds **15** and **16**—were found to be the least active compounds in the series. This less potency may be due to the non-availability of the nitrogen electron lone pair in the pyridine moiety. From the whole study it has been concluded that position, nature and number of the substituents on the phenyl ring play an important role in the inhibitory potential of the synthesized analogs.

### 2.3. Molecular Docking Study

The concentration inhibition IC_50_ values of indole based oxadiazole synthesized derivatives as β-glucoronidase inhibitors are presented in Table 1. From Table 1, it is evident that the inhibitory activity of the synthesized derivatives depends mainly on structural factors such as the type, number and position of the functional group on the phenyl ring of the synthesized derivatives. According to inhibitory IC_50_ values (Table 1) the synthesized derivatives may be classified into three groups: Highly active group with low IC_50_ values (e.g., **6** and **7**), moderate active group (e.g., **4** and **5**) and a low active group (e.g., **1** and **2**). For a better understanding of the experimental results and to emphasize the effects of type, number, and relative position of substituted groups on β-glucoronidase inhibition by the tilted compounds, molecular docking study has been performed to shed light on the established binding modes of eight selected compounds (**1** and **3**–**9**) to the closest residues in the active site of β-glucoronidase enzyme. Table 2 summarized (i) the calculated binding energies of the stable complex’s ligand-β-glucoronidase, (ii) number of established intermolecular hydrogen bonding between the synthesized compounds (**1** and **3**–**9**) and amino acid residues into the active site of β-glucoronidase, and (iii) number of closest residues surrounded the docked compounds (**1** and **3**–**9**) within the active binding site of β-glucoronidase.

**Figure 1:**
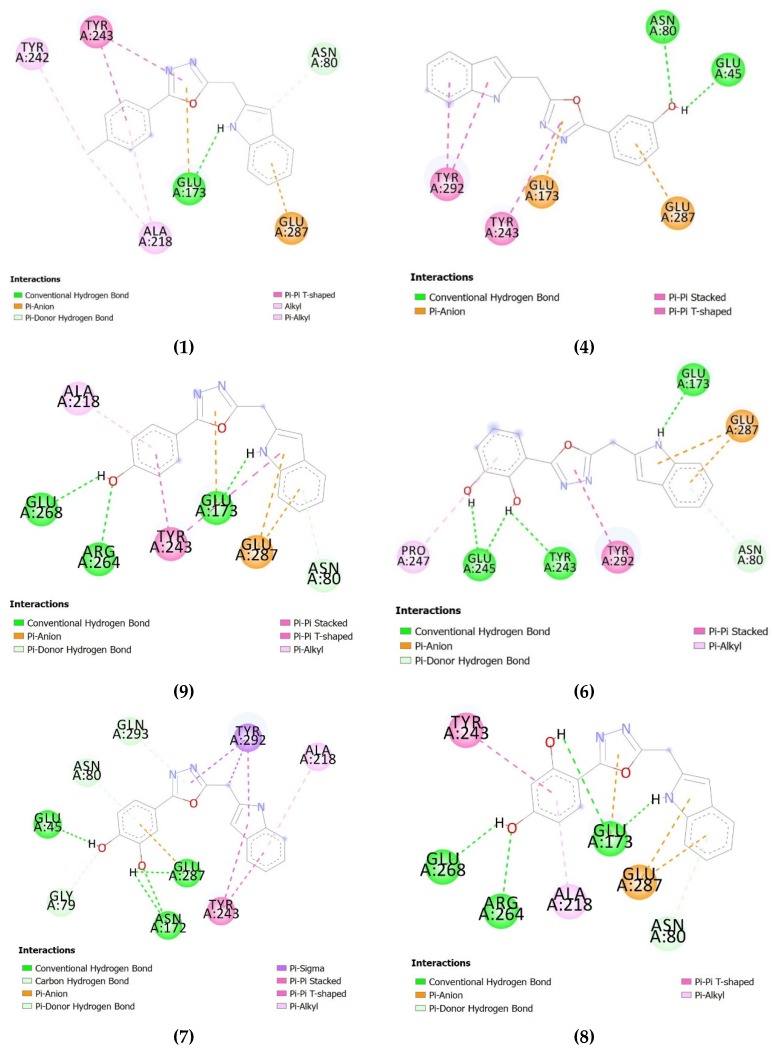
2D closest interactions between active site residues of β-glucoronidase and synthesized compounds **1**, **4**, **9**, **6**, **7**, and **8**.

Binding energies of the docked selected compounds (Table 2) within the active site β-glucoronidase show negative binding energies, which indicates the ability of the docked compound to inhibit β-glucoronidase, and that the inhibition is spontaneous. The docking results in Table 2 and Figure 1 showed that the activity of synthesized compounds may return to the number of hydrogen bonding established between functional groups of the docked ligands and amnio acid residues of the β-glucoronidase into its binding site. However, the binding energy of the formed complex between the docked compounds and β-glucoronidase, and the number of closest residues to the docked ligands has no significant contribution on the inhibitory activity of the titled compounds (Table 2 and Figure 1). The lower activity of **1** compared with **3**–**6** is mainly returned to the nature of the substituted groups in the aromatic ring (Scheme 1). The Compound **1** is substitute with a methyl group, while **3**–**6** are substituted with hydroxyl groups. Docking results show that the methyl group in **1** form alkyl and Pi-Alkyl weak interactions with ALA A 218, TYR A 242, and TYR A 243 residues into the binding site of β-glucoronidase, while in **3**–**6** the substituted hydroxyl groups forms strong hydrogen bonding interactions with amino acid residues into the binding site of β-glucoronidase (Figure 1).

The aromatic ring in **5**–**8** is substituted with two hydroxyl groups, while in **3**, **4**, and **9** is substituted only with one hydroxyl group. It is obvious from docking results that the increase of hydroxyl groups increases the number of hydrogen bonds that can be established with amino acid residues into the binding site of β-glucoronidase (Figure 1). For instance, in the case of the active Compound **6**, four hydrogen bonding are established between residues of β-glucoronidase and Compound **6**, while in 4 only two hydrogen bonds are formed (Figure 1). In **6**, the strongest hydrogen bond is formed between GLU173 amino acid and the hydrogen atom of NH of indole ring with a distance of 1.89 Å. The second strongest hydrogen bond is relatively weaker than the first one and is established between the hydrogen atom of hydroxyl of catechol oxygen in the meta position of the catechol group and GLU245 with a distance of 1.90 Å. The third strongest hydrogen bond is relatively weaker than the two first ones, and is established between the hydrogen atom of hydroxyl of catechol oxygen in *ortho* position of catechol group and GLU245 with a distance of 2.02 Å. The fourth hydrogen bond is relatively weak than the previous ones, and it is established between TYR243 amino acid and the hydrogen atom of hydroxyl of catechol oxygen in the *ortho* position of the catechol group with a distance of 2.90 Å. In case of Compound **9** with only one hydroxyl groups, three hydrogen bonding are established in the complex formed between **9** and β-glucoronidase, while for the synthesized Compound **1** only a weaker hydrogen bond formed, which is established between GLU173 amino acid and the hydrogen atom of NH of indole with a distance of 2.30 Å. As mentioned above the position of the substituted groups on the aromatic ring may have a strong influence on β-glucoronidase inhibition. Compound **7** is substituted with two hydroxyl groups on *meta* and *para* position, while **8** the two hydroxyl groups are substituted in *ortho* and *para* position. The higher activity of **7** compared with **8** may refer to the number of hydrogen bonds that are formed in **7** compared with **8**. Indeed, the two hydroxyl groups in **7** forms four hydrogen bonds with amino acids of β-glucoronidase, while in **8** the two hydroxyl groups formed only three hydrogen bonding with amino acid residues of β-glucoronidase.

## 3. Materials and Methods

### 3.1. Instruments

NMR experiments were done on Avance Bruker AM 300 MHz (Wissembourg, Switzerland). Electron impact mass spectra (EI–MS) were recorded on a Finnigan MAT-311A (Bremen, Germany). Thin layer chromatography (TLC) was performed on pre-coated silica gel aluminum plates (Kieselgel 60, 254, E. Merck, Darmstadt, Germany). Chromatograms were visualized by UV at 254 and 365 nm.

### 3.2. Molecular Docking Details

To determine the binding modes between the active site residues of β-glucuronidase and docked selected synthesized indole based oxadiazole derivatives (**1** and **3**–**9**), molecular docking has been performed by using the Lamarckian genetic algorithm as applied in the Autodock package [37]. The 3D structure of β-glucuronidase and the original docked ligand *N*-alkyl cyclophellitol aziridine were downloaded from the RCSB data bank website (PDB code 5G0Q) [38]. After removing the water molecules, Kollman charge and polar hydrogen atoms have been added to the extracted receptor structure by manipulating the AutoDock Tools 4.2 (The Scripps Research Institute, La Jolla, CA, USA). The re-docking of the original ligand *N*-alkyl cyclophellitol aziridine into the active site of β-glucuronidase is well reproduced with an RMSD value lower than 2 Å. **1** and **3**–**9** structural geometries were optimized using Merck molecular force field 94 as implemented in Hyperchem Software (HyperChem Release 7.52, Hypercube Inc., Gainesville, FL, USA, 2002), and saved as protein data bank (pdf) files. For each docked ligand, non-polar hydrogen’s were merged and rotatable bonds were defined. Docking run was performed by the Lamarckian genetic algorithm, with 500 as a total number of run for originated ligand and 100 run for **1**, **4**, and **6** derivatives. A total population of 150 individuals with 27,000 generations and 250,000 energy evaluations were utilized in each particular run. Operator weights for crossover, mutation, and elitism were set to 0.8, 0.02, and 1, respectively. The binding site was defined using a grid of 40 × 40 × 40 points each with a grid spacing of 0.375 Å. Intel (R) core (M) 15-3770 CPU @ 3.40 GHz workstation is used to carry out docking calculation.

### 3.3. Synthesis Methyl 2-(1H-indol-2-yl)acetate

The methyl 2-(1*H*-indol-2-yl) acetate (10 g, 52.88 mmol) was treated with hydrazine (15 mL) in ethanol (50 mL) for 6 h. The excess hydrazine and ethanol were evaporated to get crude product then recrystallized by ethanol to yield 2-(1*H*-indol-2-yl) acetohydrazide with 90% (9.01 g, 47.76 mmol) produced [29].

### 3.4. General Procedure Indole Based 2,5-Disubstituted-1,3,4-Oxadiazoles (1–22)

A combination of methyl 2-(1*H*-indol-2-yl) acetohydrazide (0.3 mmol) and several aromatic acid (0.33 mmol) in POCl_3_ (7 mL) was reacted for 4–6 h. The mixture left for cooling once confirmed reaction completion by TLC then poured into cold water. The reaction mixture was treated with NaHCO_3_ solution and the subsequent solid formed was filtered, dried, and crystallized in ethanolic solution [39].

#### 3.4.1. 2-((1*H*-indol-2-yl) methyl)-5-(*p*-tolyl)-1,3,4-oxadiazole

Yield 84%, m.p. 304–305 °C; ^1^H-NMR (500 MHz, DMSO-*d*_6_): δ 11.67 (s, 1H, NH), 7.97 (d, *J* = 7.5 Hz, 2H, Ar), 7.51 (d, *J* = 7.1 Hz, 1H, Ar), 7.28 (d, *J* = 6.8 Hz, 1H, Ar), 7.26 (d, *J* = 7.4 Hz, 2H, Ar), 7.10 (m, 1H, Ar), 7.06 (m, 1H, Ar), 6.22 (s, 1H, Ar), 3.85 (s, 2H, CH_2_), 2.34 (s, 3H, CH_3_), ^13^C-NMR (125 MHz, DMSO-*d*_6_): δ 166.4, 164.5, 136.5, 135.6, 131.7, 128.1, 127.4, 127.4, 126.3, 126.3, 121.7, 120.7, 120.2, 119.8, 111.1, 101.5, 31.0, 21.3, HREI-MS: *m*/*z* calcd. for C_18_H_15_N_3_O [M]^+^ 289.1215; Found 289.1211

#### 3.4.2. 2-((1*H*-indol-2-yl) methyl)-5-(*o*-tolyl)-1,3,4-oxadiazole

Yield 74%, m.p. 300–301 °C; ^1^H-NMR (500 MHz, DMSO-*d_6_*): δ 11.67 (s, 1H, NH), 7.73 (d, *J* = 7.5 Hz, 7.72 (d, *J* = 7.5 Hz, 1H, Ar), 7.51 (d, *J* = 7.1 Hz, 1H, Ar), 7.40 (d, *J* = 7.1 Hz, 1H, Ar), 7.35 (m, 1H, Ar), 7.35 (d, *J* = 7.0 Hz, 1H, Ar), 7.28 (d, *J* = 6.8 Hz, 1H, Ar), 7.10 (m, 1H, Ar), 7.06 (m, 1H, Ar), 6.22 (s, 1H, Ar), 3.85 (s, 2H, CH_2_), 1H, Ar), 2.57 (s, 3H, CH_3_), ^13^C-NMR (125 MHz, DMSO-*d_6_*): δ 166.4, 164.5, 137.2, 136.9, 136.5, 135.6, 129.5, 128.6, 128.1, 127.4, 126.2, 121.7, 120.7, 119.8, 111.1, 101.5, 31.0, 18.7, HREI-MS: *m*/*z* calcd. for C_18_H_15_N_3_O [M]^+^ 289.1215; Found 289.1210.

#### 3.4.3. 2-(5-((1*H*-indol-2-yl) methyl)-1,3,4-oxadiazol-2-yl) phenol

Yield 69%, m.p. 314–315 °C; ^1^H-NMR (500 MHz, DMSO-*d_6_*): δ 11.67 (s, 1H, NH), 7.60 (d, *J* = 7.1 Hz, 1H, Ar), 7.51 (d, *J* = 7.1 Hz, ^1^H, Ar), 7.32 (m, 1H, Ar), 7.28 (d, *J* = 6.8 Hz, 1H, Ar), 7.10 (m, 1H, Ar), 7.06 (m, 1H, Ar), 7.00 (d, *J* = 6.7 Hz, 1H, Ar), 7.06 (m, 1H, Ar), 6.22 (s, 1H, Ar), 3.85 (s, 2H, CH_2_), ^13^C-NMR (125 MHz, DMSO-*d_6_*): δ 166.4, 164.5, 157.4, 136.5, 135.6, 130.1, 128.1, 126.3, 121.8, 121.7, 120.7, 119.8, 117.8, 111.1, 108.0, 101.5, 31.0, HREI-MS: *m*/*z* calcd. for C_17_H_13_N_3_O_2_ [M]^+^ 291.1008; Found 291.1004

#### 3.4.4. 3-(5-((1*H*-indol-2-yl) methyl)-1,3,4-oxadiazol-2-yl) phenol

Yield 79%, m.p. 311–312 °C; ^1^H-NMR (500 MHz, DMSO-*d_6_*): δ 11.67 (s, 1H, NH), 7.61 (d, *J* = 7.1 Hz, 1H, Ar), 7.51 (d, *J* = 7.1 Hz, 1H, Ar), 7.28 (d, *J* = 6.8 Hz, 1H, Ar), 7.27 (m, 1H, Ar), 7.14 (s, 1H, Ar), 7.10 (m, 1H, Ar), 7.06 (m, 1H, Ar), 6.82 (d, *J* = 6.3 Hz, 1H, Ar), 6.22 (s, 1H, Ar), 3.85 (s, 2H, CH_2_), ^13^C-NMR (125 MHz, DMSO-*d6*): δ 166.4, 164.5, 157.5, 136.5, 135.6, 130.6, 128.1, 127.5, 121.7, 120.7, 120.1, 119.8, 115.9, 112.9, 111.1, 101.5, 31.0, HREI-MS: *m*/*z* calcd. for C_17_H_13_N_3_O_2_ [M]^+^ 291.1008; Found 291.1003

#### 3.4.5. 2-(5-((1*H*-indol-2-yl) methyl)-1,3,4-oxadiazol-2-yl) benzene-1,4-diol

Yield 70%, m.p. 319–320 °C; ^1^H-NMR (500 MHz, DMSO-*d_6_*): δ 11.67 (s, 1H, NH), 7.51 (d, *J* = 7.1 Hz, 1H, Ar), 7.28 (d, *J* = 6.8 Hz, 1H, Ar), 7.10 (m, 1H, Ar), 7.06 (m, 1H, Ar), 7.04 (d, *J* = 6.7 Hz, 1H, Ar), 6.97 (s, 1H, Ar), 6.62 (d, *J* = 6.6 Hz, 1H, Ar), 6.22 (s, 1H, Ar), 3.85 (s, 2H, CH_2_), ^13^C-NMR (125 MHz, DMSO-*d6*): δ 166.4, 164.5, 150.1, 150.0, 136.5, 135.6, 128.1, 121.7, 120.7, 119.8, 117.8, 117.3, 114.3, 113.5, 111.1, 101.5, 31.0, HREI-MS: *m*/*z* calcd. for C_17_H_13_N_3_O_3_ [M]^+^ 307.0957; Found 307.0951

#### 3.4.6. 3-(5-((1*H*-indol-2-yl) methyl)-1,3,4-oxadiazol-2-yl) benzene-1,2-diol

Yield 73%, m.p. 317–318 °C; 1H-NMR (500 MHz, DMSO-*d*_6_): δ 11.67 (s, 1H, NH), 7.51 (d, *J* = 7.1 Hz, 1H, Ar), 7.28 (d, *J* = 6.8 Hz, 1H, Ar), 7.16 (d, *J* = 6.9 Hz, 1H, Ar), 7.10 (m, 1H, Ar), 7.06 (m, 1H, Ar), 6.96 (d, *J* = 6.5 Hz, Ar), 6.83 (dd, J = 7.8, 3.5 Hz, Ar), 6.22 (s, 1H, Ar), 3.85 (s, 2H, CH_2_), ^13^C-NMR (125 MHz, DMSO-*d_6_*): δ 166.4, 164.5, 145.6, 143.9, 136.5, 135.6, 130.7, 128.1, 123.2, 121.7, 120.7, 119.8, 117.3, 113.5, 111.1, 101.5, 31.0, HREI-MS: *m*/*z* calcd. for C_17_H_13_N_3_O_3_ [M]^+^ 307.0957; Found 307.0952

#### 3.4.7. 4-(5-((1*H*-indol-2-yl) methyl)-1,3,4-oxadiazol-2-yl) benzene-1,2-diol

Yield 85%, m.p. 316–317 °C; ^1^H-NMR (500 MHz, DMSO-*d_6_*): δ 11.67 (s, 1H, NH), 7.51 (d, *J* = 7.1 Hz, 1H, Ar), 7.41 (d, *J* = 7.3 Hz, Ar), 7.28 (d, *J* = 6.8 Hz, 1H, Ar), 7.10 (m, 1H, Ar), 7.06 (m, 1H, Ar), 6.83 (d, *J* = 6.4 Hz, Ar), 6.37 (s, 1H, Ar), 6.22 (s, 1H, Ar), 3.85 (s, 2H, CH_2_), ^13^C-NMR (125 MHz, DMSO-*d_6_*): δ 166.4, 164.5, 147.3, 145.9, 136.5, 135.6, 128.1, 121.7, 120.7, 120.1, 119.8, 116.2, 114.3, 111.1, 108.9, 101.5, 31.0, HREI-MS: *m*/*z* calcd. for C_17_H_13_N_3_O_3_ [M]^+^ 307.0957; Found 307.0952

#### 3.4.8. 4-(5-((1*H*-indol-2-yl) methyl)-1,3,4-oxadiazol-2-yl) benzene-1,3-diol

Yield 83%, m.p. 318–319 °C; ^1^H-NMR (500 MHz, DMSO-*d_6_*): δ 11.67 (s, 1H, NH), 7.51 (d, *J* = 7.1 Hz, 1H, Ar), 7.43 (d, *J* = 7.1 Hz, 1H, Ar), 7.28 (d, *J* = 6.8 Hz, 1H, Ar), 7.10 (m, 1H, Ar), 7.06 (m, 1H, Ar), 6.42 (d, *J* = 6.2 Hz, 1H, Ar), 6.33 (s, 1H, Ar), 6.22 (s, 1H, Ar), 3.85 (s, 2H, CH_2_), ^13^C-NMR (125 MHz, DMSO-*d_6_*): δ 166.4, 164.5, 159.9, 156.6, 136.5, 135.6, 130.3, 128.1, 121.7, 120.7, 119.8, 111.1, 109.0, 105.6, 101.5, 100.6, 31.0, HREI-MS: *m*/*z* calcd. for C_17_H_13_N_3_O_3_ [M]^+^ 307.0957; Found 307.0951

#### 3.4.9. 4-(5-((1*H*-indol-2-yl) methyl)-1,3,4-oxadiazol-2-yl) phenol

Yield 70%, m.p. 310–311 °C; ^1^H-NMR (500 MHz, DMSO-*d_6_*): δ 11.67 (s, 1H, NH), 7.85 (d, *J* = 7.4 Hz, 2H, Ar), 7.51 (d, *J* = 7.1 Hz, 1H, Ar), 7.28 (d, *J* = 6.8 Hz, 1H, Ar), 7.10 (m, 1H, Ar), 7.06 (m, 1H, Ar), 6.91 (d, *J* = 6.7 Hz, 2H, Ar), 6.22 (s, 1H, Ar), 3.85 (s, 2H, CH_2_), ^13^C-NMR (125 MHz, DMSO-*d_6_*): δ 166.4, 164.5, 158.5, 136.5, 135.6, 128.1, 121.7, 120.7, 119.8, 118.7, 116.4, 116.4, 116.3, 116.3, 111.1, 101.5, 31.0, HREI-MS: *m*/*z* calcd. for C_17_H_13_N_3_O_2_ [M]^+^ 291.1008; Found 291.1003

#### 3.4.10. 2-((1*H*-indol-2-yl) methyl)-5-(2-nitrophenyl)-1,3,4-oxadiazole

Yield 65%, m.p. 307–308 °C; ^1^H-NMR (500 MHz, DMSO-*d_6_*): δ 11.67 (s, 1H, NH), 8.03 (d, *J* = 7.8 Hz, 1H, Ar), 8.00 (d, *J* = 7.4 Hz, 1H, Ar), 7.89 (m, 1H, Ar), 7.72 (m, 1H, Ar), 7.51 (d, *J* = 7.1 Hz, 1H, Ar), 7.28 (d, *J* = 6.8 Hz, 1H, Ar), 7.10 (m, 1H, Ar), 7.06 (m, 1H, Ar), 6.22 (s, 1H, Ar), 3.85 (s, 2H, CH_2_), ^13^C-NMR (125 MHz, DMSO-*d6*): δ 166.4, 164.5, 146.9, 136.5, 135.6, 135.3, 131.6, 129.6, 128.4, 128.1, 124.4, 121.7, 120.7, 119.8, 111.1, 101.5, 31.0, HREI-MS: *m*/*z* calcd. for C_17_H_12_N_4_O_3_ [M]^+^ 320.0909; Found 320.0904

#### 3.4.11. 2-((1*H*-indol-2-yl) methyl)-5-(3-nitrophenyl)-1,3,4-oxadiazole

Yield 89%, m.p. 303–304 °C; ^1^H-NMR (500 MHz, DMSO-*d_6_*): δ 11.67 (s, 1H, NH), 8.60 (s, 1H, Ar), 8.44 (d, *J* = 8.0 Hz, 1H, Ar), 8.32 (d, *J* = 7.9 Hz, 1H, Ar), 7.84 (m, 1H, Ar), 7.51 (d, *J* = 7.1 Hz, 1H, Ar), 7.28 (d, *J* = 6.8 Hz, 1H, Ar), 7.10 (m, 1H, Ar), 7.06 (m, 1H, Ar), 6.22 (s, 1H, Ar), 3.85 (s, 2H, CH_2_), ^13^C-NMR (125 MHz, DMSO-*d6*): δ 166.4, 164.5, 146.9, 136.5, 135.6, 135.3, 131.6, 129.6, 128.4, 128.1, 124.4, 121.7, 120.7, 119.8, 111.1, 101.5, 31.0, HREI-MS: *m*/*z* calcd. for C_17_H_12_N_4_O_3_ [M]^+^ 320.0909; Found 320.0903

#### 3.4.12. 2-((1*H*-indol-2-yl) methyl)-5-(4-nitrophenyl)-1,3,4-oxadiazole

Yield 71%, m.p. 309–310 °C; ^1^H-NMR (500 MHz, DMSO-*d_6_*): δ 11.67 (s, 1H, NH), 8.41 (d, *J* = 8.2 Hz, 2H, Ar), 8.23 (d, *J* = 8.1 Hz, 2H, Ar), 7.51 (d, *J* = 7.1 Hz, 1H, Ar), 7.28 (d, *J* = 6.8 Hz, 1H, Ar), 7.10 (m, 1H, Ar), 7.06 (m, 1H, Ar), 6.22 (s, 1H, Ar), 3.85 (s, 2H, CH_2_), 13C-NMR (125 MHz, DMSO-*d_6_*): δ 166.4, 164.5, 146.9, 136.5, 135.6, 135.3, 131.6, 129.6, 128.4, 128.1, 124.4, 121.7, 120.7, 119.8, 111.1, 101.5, 31.0, HREI-MS: *m*/*z* calcd. for C_17_H_12_N_4_O_3_ [M]^+^ 320.0909; Found 320.0905

#### 3.4.13. 2-(5-((1*H*-indol-2-yl) methyl)-1,3,4-oxadiazol-2-yl)-4-methoxyphenol

Yield 86%, m.p. 313–314 °C; ^1^H-NMR (500 MHz, DMSO-*d_6_*): δ 11.67 (s, 1H, NH), 7.51 (d, *J* = 7.1 Hz, 1H, Ar), 7.28 (d, *J* = 6.8 Hz, 1H, Ar), 7.12 (s, 1H, Ar), 7.10 (m, 1H, Ar), 7.06 (m, 1H, Ar), 6.79 (d, *J* = 6.3 Hz, 1H, Ar), 6.72 (d, *J* = 6.2 Hz, 1H, Ar), 6.22 (s, 1H, Ar), 3.85 (s, 2H, CH_2_), 3.81 (s, 3H, CH_3_), ^13^C-NMR (125 MHz, DMSO-*d_6_*): δ 166.4, 164.5, 153.7, 149.7, 136.5, 135.6, 128.1, 121.7, 120.7, 119.8, 117.4, 115.7, 113.1, 112.7, 111.1, 101.5, 55.8, 31.0, HREI-MS: *m*/*z* calcd. for C_18_H_15_N_3_O_3_ [M]^+^ 321.1113; Found 321.1110

#### 3.4.14. 2-((1*H*-indol-2-yl) methyl)-5-(3-bromo-4-fluorophenyl)-1,3,4-oxadiazole

Yield 74%, m.p. 290–291 °C; ^1^H-NMR (500 MHz, DMSO-*d_6_*): δ 11.67 (s, 1H, NH), 8.23 (d, *J* = 7.8 Hz, 1H, Ar), 7.51 (d, *J* = 7.1 Hz, 1H, Ar), 7.43 (s, 1H, Ar), 7.29 (d, *J* = 7.2 Hz, 1H, Ar), 7.28 (d, *J* = 6.8 Hz, 1H, Ar), 7.10 (m, 1H, Ar), 7.06 (m, 1H, Ar), 6.22 (s, 1H, Ar), 3.85 (s, 2H, CH_2_), ^13^C-NMR (125 MHz, DMSO-*d_6_*): δ 166.4, 165.4, 164.5, 136.5, 135.6, 134.7, 128.1, 128.1, 123.9, 121.7, 120.7, 119.8, 118.2, 111.1, 110.6, 101.5, 31.0, HREI-MS: *m*/*z* calcd. for C_17_H_11_BrFN_3_O [M]^+^ 371.0070; Found 371.0065

#### 3.4.15. 2-((1*H*-indol-2-yl) methyl)-5-(3-chlorophenyl)-1,3,4-oxadiazole

Yield 81%, m.p. 286–287 °C; ^1^H-NMR (500 MHz, DMSO-*d_6_*): δ 11.67 (s, 1H, NH), 7.97 (s, 1H, Ar), 7.93 (d, *J* = 7.3 Hz, 1H, Ar), 7.51 (d, *J* = 7.1 Hz, 1H, Ar), 7.48 (m, 1H, Ar), 7.46 (d, *J* = 7.1 Hz, 1H, Ar), 7.28 (d, *J* = 6.8 Hz, 1H, Ar), 7.10 (m, 1H, Ar), 7.06 (m, 1H, Ar), 6.22 (s, 1H, Ar), 3.85 (s, 2H, CH_2_), ^13^C-NMR (125 MHz, DMSO-*d_6_*): δ 166.4, 164.5, 136.5, 135.6, 134.8, 129.5, 128.8, 128.1, 127.5, 127.4, 125.6, 121.7, 120.7, 119.8, 111.1, 101.5, 31.0, HREI-MS: *m*/*z* calcd. for C_17_H_12_ClN_3_O [M]^+^ 309.0669; Found 309.0663

#### 3.4.16. 2-((1*H*-indol-2-yl) methyl)-5-(pyridin-3-yl)-1,3,4-oxadiazole

Yield 68%, m.p. 279–280 °C; ^1^H-NMR (500 MHz, DMSO-*d_6_*): δ 11.67 (s, 1H, NH), 9.24 (s, 1H, Ar), 8.70 (d, *J* = 8.4 Hz, 1H, Ar), 7.51 (d, *J* = 7.1 Hz, 1H, Ar), 8.42 (d, *J* = 7.8 Hz, 1H, Ar), 7.57 (m, 1H, Ar), 7.28 (d, *J* = 6.8 Hz, 1H, Ar), 7.10 (m, 1H, Ar), 7.06 (m, 1H, Ar), 6.22 (s, 1H, Ar), 3.85 (s, 2H, CH_2_), ^13^C-NMR (125 MHz, DMSO-*d_6_*): δ 166.4, 164.5, 152.7, 147.9, 136.5, 135.6, 134.0, 128.1, 124.4, 124.0, 121.7, 120.7, 119.8, 111.1, 101.5, 31.0, HREI-MS: *m*/*z* calcd. for C_16_H_12_N_4_O [M]^+^ 276.1011; Found 276.1006

#### 3.4.17. 2-((1*H*-indol-2-yl) methyl)-5-(pyridin-4-yl)-1,3,4-oxadiazole

Yield 80%, m.p. 284–285 °C; ^1^H-NMR (500 MHz, DMSO-*d_6_*): δ 11.67 (s, 1H, NH), 8.75 (d, *J* = 8.2 Hz, 2H, Ar), 7.99 (d, *J* = 7.3 Hz, 2H, Ar), 7.51 (d, *J* = 7.1 Hz, 1H, Ar), 7.28 (d, *J* = 6.8 Hz, 1H, Ar), 7.10 (m, 1H, Ar), 7.06 (m, 1H, Ar), 6.22 (s, 1H, Ar), 3.85 (s, 2H, CH_2_), ^13^C-NMR (125 MHz, DMSO-*d_6_*): δ 166.4, 164.5, 152.7, 147.9, 136.5, 135.6, 134.0, 128.1, 124.4, 124.0, 121.7, 120.7, 119.8, 111.1, 101.5, 31.0, HREI-MS: *m*/*z* calcd. for C_16_H_12_N_4_O [M]^+^ 276.1011; Found 276.1007

#### 3.4.18. 2-((1*H*-indol-2-yl) methyl)-5-(2-fluorophenyl)-1,3,4-oxadiazole

Yield 72%, m.p. 294–295 °C; ^1^H-NMR (500 MHz, DMSO-*d_6_*): δ 11.67 (s, 1H, NH), 8.26 (d, *J* = 7.3 Hz, 1H, Ar), 7.71 (m, 1H, Ar),7.51 (d, *J* = 7.1 Hz, 1H, Ar), 7.49 (m, 2H, Ar), 7.28 (d, *J* = 6.8 Hz, 1H, Ar), 7.10 (m, 1H, Ar), 7.06 (m, 1H, Ar), 6.22 (s, 1H, Ar), 3.85 (s, 2H, CH_2_), ^13^C-NMR (125 MHz, DMSO-*d_6_*): δ 166.4, 164.5, 158.3, 136.5, 135.6, 130.3, 129.1, 128.1, 124.8, 123.5, 121.7, 120.7, 119.8, 114.7, 111.1, 101.5, 31.0, HREI-MS: *m*/*z* calcd. for C_17_H_12_FN_3_O [M]^+^ 293.0964; Found 293.0961

#### 3.4.19. 2-((1*H*-indol-2-yl) methyl)-5-(3-fluorophenyl)-1,3,4-oxadiazole

Yield 87%, m.p. 291–292 °C; ^1^H-NMR (500 MHz, DMSO-*d_6_*): δ 11.67 (s, 1H, NH), 7.82 (d, *J* = 7.2 Hz, 1H, Ar), 7.52 (s, 1H, Ar), 7.51 (d, *J* = 7.1 Hz, 1H, Ar), 7.50 (m, 1H, Ar), 7.28 (d, *J* = 6.8 Hz, 1H, Ar), 7.22 (d, *J* = 63 Hz, 1H, Ar), 7.10 (m, 1H, Ar), 7.06 (m, 1H, Ar), 6.22 (s, 1H, Ar), 3.85 (s, 2H, CH_2_), ^13^C-NMR (125 MHz, DMSO-*d_6_*): δ 166.4, 164.5, 162.0, 136.5, 135.6, 128.1, 127.7, 127.5, 123.1, 121.7, 120.7, 119.8, 115.9, 115.5, 111.1, 101.5, 31.0, HREI-MS: *m*/*z* calcd. for C_17_H_12_FN_3_O [M]^+^ 293.0964; Found 293.0960

#### 3.4.20. 2-((1*H*-indol-2-yl) methyl)-5-(4-fluorophenyl)-1,3,4-oxadiazole

Yield 83%, m.p. 300–301 °C; ^1^H-NMR (500 MHz, DMSO-*d_6_*): δ 11.67 (s, 1H, NH), 8.29 (d, *J* = 7.5 Hz, 2H, Ar), 7.51 (d, *J* = 7.1 Hz, 1H, Ar), 7.44 (d, *J* = 7.0 Hz, 2H, Ar), 7.28 (d, *J* = 6.8 Hz, 1H, Ar), 7.10 (m, 1H, Ar), 7.06 (m, 1H, Ar), 6.22 (s, 1H, Ar), 3.85 (s, 2H, CH_2_), ^13^C-NMR (125 MHz, DMSO-*d_6_*): δ 166.4, 164.5, 162.9, 136.5, 135.6, 129.1, 129.1, 128.1, 121.7, 121.7, 120.7, 119.8, 116.0, 116.0, 111.1, 101.5, 31.0, HREI-MS: *m*/*z* calcd. for C_17_H_12_FN_3_O [M]^+^ 293.0964; Found 293.0962

#### 3.4.21. 2-((1*H*-indol-2-yl) methyl)-5-(2-chlorophenyl)-1,3,4-oxadiazole

Yield 72%, m.p. 280–281 °C; ^1^H-NMR (500 MHz, DMSO-*d_6_*): δ 11.67 (s, 1H, NH), 7.71 (d, *J* = 7.2 Hz, 1H, Ar), 7.61 (d, *J* = 7.0 Hz, 1H, Ar), 7.51 (d, *J* = 7.1 Hz, 1H, Ar), 7.38 (m, 1H, Ar), 7.37 (m, 1H, Ar), 7.28 (d, *J* = 6.8 Hz, 1H, Ar), 7.10 (m, 1H, Ar), 7.06 (m, 1H, Ar), 6.22 (s, 1H, Ar), 3.85 (s, 2H, CH_2_), ^13^C-NMR (125 MHz, DMSO-*d_6_*): δ 166.4, 164.5, 136.9, 136.5, 135.6, 132.2, 130.1, 129.3, 128.9, 128.1, 127.3, 121.7, 120.7, 119.8, 111.1, 101.5, 31.0, HREI-MS: *m*/*z* calcd. for C_17_H_12_ClN_3_O [M]^+^ 309.0669; Found 309.0666

#### 3.4.22. 2-((1*H*-indol-2-yl) methyl)-5-(4-chlorophenyl)-1,3,4-oxadiazole

Yield 77%, m.p. 287–288 °C; ^1^H-NMR (500 MHz, DMSO-*d_6_*): δ 11.67 (s, 1H, NH), 7.71 (d, *J* = 7.2 Hz, 2H, Ar), 7.53 (d, *J* = 6.9 Hz, 2H, Ar), 7.51 (d, *J* = 7.1 Hz, 1H, Ar), 7.28 (d, *J* = 6.8 Hz, 1H, Ar), 7.10 (m, 1H, Ar), 7.06 (m, 1H, Ar), 6.22 (s, 1H, Ar), 3.85 (s, 2H, CH_2_), ^13^C-NMR (125 MHz, DMSO-*d_6_*): δ 166.4, 164.5, 136.5, 135.6, 134.3, 129.3, 129.3, 128.9, 128.9, 128.1, 124.2, 121.7, 120.7, 119.8, 111.1, 101.5, 31.0, HREI-MS: *m*/*z* calcd. for C_17_H_12_ClN_3_O [M]^+^ 309.0669; Found 309.0669

### 3.5. β-Glucuronidase Assay

β-glucuronidase activity was determined in accordance to method used [40] by measuring absorbance at 405 nm of p-nitrophenol formed substrate by spectrophotometric method. 250 µL was the volume of total reaction. Reaction mixture containing 5 µL of test compound solution, 185 µL of 0.1M acetate buffer, and 10 µL of enzyme solution were incubated for 30 min at 37 °C. At 405 nm the plates were recorded on multiplate reader (SpectaMax plus 384) after the addition of 50 µL of 0.4mM p-nitrophenyl-β-d-glucuronide. Experiments were performed for triplicate [41]. To avoid precipitation, compound concentrations were decreased, and the volume of reaction was increased (200 µL). Precipitation probability was less thus addition of detergents was not needed.

## 4. Conclusions

β-glucuronidase is a member of the enzyme lysosomal glycosidase. The function of this enzyme is to catalyze the extracellular matrix of cancer and normal cells and also catalyze glycosaminoglycans of the cell membrane. This catalysis is important for cancer cell proliferation, invasion and metastasis. Liver cancer, colon carcinoma, and neoplasm bladder are caused by the increase level of β-glucuronidase activity. The most valuable structures are indole and oxadiazole which has gain immense attention because of its pharmacological behavior and display many biological properties. The synthesized analogs (**1**–**22**), indole based oxadiazole analogs were evaluated for their inhibitory potential against the β-glucuronidase enzyme. Majority of the compounds showed potent inhibitory potential with IC_50_ values ranging from 0.9 ± 0.01 to 46.4 ± 0.9 µM when compared with the standard drug d-saccharic acid 1,4 lactonec (IC_50_ = 48.1 ± 1.2 µM). Structural activity relationship (SAR) has been established for all synthesized compounds. Molecular docking of **1**, **4**, and **6** into the active site of β-glucuronidase showed that the β-glucuronidase inhibition by the tilted compounds is thermodynamically favorable and increases with the number of hydrogens bonding intermolecular interactions.

## Figures and Tables

**Table 1:** Synthesis of indole based oxadiazoles (**1**–**22**) and their in vitro β-glucuronidase inhibition.

S. No.		Yield (%)	IC_50_ (*μ*M ± SEM ^a^)	S. No.		Yield (%)	IC_50_ (*μ*M ± SEM ^a^)
1	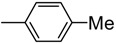		36.4 ± 0.6	12	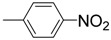	90	37.3 ± 0.7
2	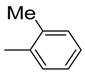		26.2 ± 0.8	13	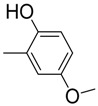	82	20.6 ± 0.5
3	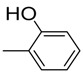		6.2 ± 0.2	14	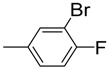	84	NA ^b^
4	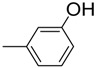	84	17.90 ± 0.4	15	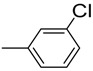	83	34.6 ± 0.7
5	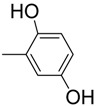	85	11.4 ± 0.30	16	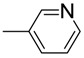	85	46.4 ± 0.9
6	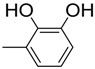	86	0.9 ± 0.01	17	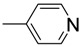	87	43.1 ± 0.8
7	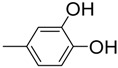	81	1.2 ± 0.01	18	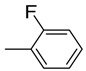	88	5.0 ± 0.1
8	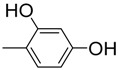	83	7.2 ± 0.10	19	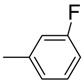	90	10.5 ± 0.2
9	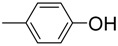	88	11.0 ± 0.4	20	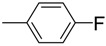	92	17.0 ± 0.4
10	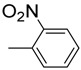	90	40.0 ± 0.7	21	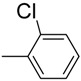	90	6.0 ± 0.2
11	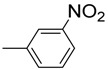	87	NA ^b^	22	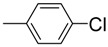	82	22.2 ± 0.5
d-saccharic acid 1,4 lactone ^c^				48.1 ± 1.2			

SEM ^a^: Standard error Mean; N.B.^b^: Not Active; d-saccharic acid 1,4 lactone ^c^: Standard Drug.

**Table 2:** Concentration inhibition IC_50_, docking binding energies and number of closest residues to the docked ligands (**1**, **4**, and **6**) within the active binding site of β-glucoronidase.

Indole Based Oxadiazole Derivatives	Free Binding Energy (kcal/mol)	H-Bonds	Number of Closest Residues to the Docked Ligand in the Active Site	IC_50_ ± SEM
**1**	−8.74	1	5	36.4 ± 0.6
**3**	−8.17	1	4	6.2 ± 0.2
**4**	−8.53	2	6	17.9 ± 0.4
**5**	−9.16	3	7	11.4 ± 0.3
**6**	−8.77	4	7	0.9 ± 0.01
**7**	−8.88	4	9	1.2 ± 0.01
**8**	−8.56	4	7	7.2 ± 0.10
**9**	−8.65	3	7	11.04 ± 0.4
